# Precise mapping of one classic and three novel *GluRIIA* mutants in *Drosophila melanogaster*

**DOI:** 10.17912/micropub.biology.000784

**Published:** 2023-06-02

**Authors:** Bhagaban Mallik, Douglas J Brusich, Georgette Heyrman, C. Andrew Frank

**Affiliations:** 1 Department of Anatomy and Cell Biology, University of Iowa, Iowa City, Iowa, United States; 2 Human Biology Department, University of Wisconsin–Green Bay, Green Bay, Wisconsin, United States; 3 Carver College of Medicine, University of Iowa, Iowa City, Iowa, United States; 4 Iowa Neuroscience Institute, University of Iowa, Iowa City, Iowa, United States

## Abstract

Mutation of the *Drosophila melanogaster* *GluRIIA* gene or pharmacological agents targeting it are commonly used to assess homeostatic synaptic function at the larval neuromuscular junction (NMJ). The commonly used mutation, *GluRIIA ^SP16^* , is a null allele created by a large and imprecise excision of a P-element which affects *GluRIIA* and multiple upstream genes. Here we mapped the exact bounds of the *GluRIIA ^SP16^* allele, refined a multiplex PCR strategy for positive identification of *GluRIIA ^SP16^* in homozygous or heterozygous backgrounds, and sequenced and characterized three new CRISPR-generated *GluRIIA* mutants. We found the three new *GluRIIA* alleles are apparent nulls that lack GluRIIA immunofluorescence signal at the 3 ^rd^ instar larval NMJ and are predicted to cause premature truncations at the genetic level. Further, these new mutants have similar electrophysiological outcomes as *GluRIIA ^SP16^* , including reduced miniature excitatory postsynaptic potential (mEPSP) amplitude and frequency compared to controls, and they express robust homeostatic compensation as evidenced by normal excitatory postsynaptic potential (EPSP) amplitude and elevated quantal content. These findings and new tools extend the capacity of the *D. melanogaster* NMJ for assessment of synaptic function.

**Figure 1. Identification of exact bounds of the GluRIIA SP16 lesion and three new CRISPR-generated GluRIIA mutants.**
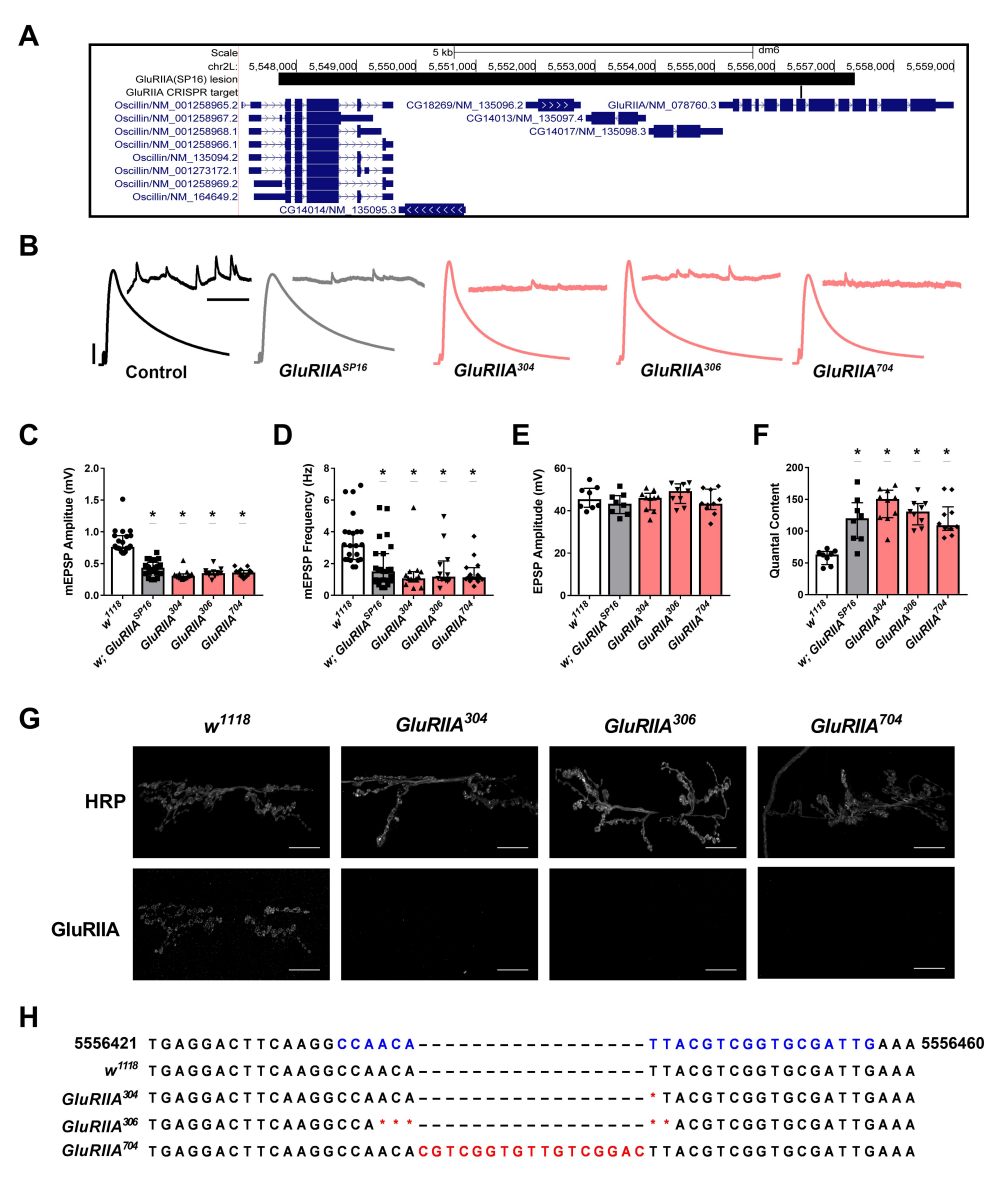
(A) The *GluRIIA* gene region is shown with the span of the *GluRIIA ^SP16^* lesion and the location of the CRISPR target site in exon 6 of *GluRIIA* . (B) Representative traces of EPSP and mEPSP electrophysiology. Horizontal and vertical scale bars for EPSP (mEPSP) traces are 50 ms (1000ms) and 10 mV (1 mV) respectively. (C) mEPSP amplitude, (D) mEPSP frequency, (E) EPSP amplitude, and (F) quantal content were assessed at the NMJ of wandering 3 ^rd^ instar animals. (C-F) Data plotted are medians with 25%-75% interquartile ranges. Each symbol represents a single muscle (n ≥ 8 muscles per condition). * = p < 0.05 by Kruskal-Wallis with Dunn’s correction for multiple comparisons of *GluRIIA* mutant lines to *w ^1118^* controls. (G) Larval NMJs were stained with HRP (top) to detect the motor nerve and anti-GluRIIA antibodies (bottom). G *luRIIA ^304^* , *GluRIIA ^306^* , and *GluRIIA ^704^* failed to generate signal for GluRIIA. (H) The reference genome (chr2L: 5,556,421 - 5,556,460) with CRISPR-target site (blue), were aligned to DNA sequencing reads. Red symbols (*) and text in CRISPR-generated lines denote deleted and inserted nucleotides respectively.

## Description

Glutamate is the major excitatory neurotransmitter of the mammalian central nervous system (CNS). Regulation of glutamate receptors is essential to normal nervous system activities and plasticity, while their perturbation contributes to various nervous system diseases. The fruit fly ( *Drosophila melanogaster* ) neuromuscular junction (NMJ) offers a readily accessible glutamatergic synapse in a genetically tractable organism (Jan and Jan 1976) . The utility of the fly NMJ has been demonstrated through findings of conserved mechanisms of synaptic function and modeling of human disease (Harris and Littleton 2015) . Ionotropic glutamate receptors at the fly NMJ are tetrameric proteins made of the essential subunits DGluRIIC, DGluRIID, DGluRIIE, and one of either DGluRIIA or DGluRIIB (Petersen et al. 1997; Diantonio et al. 1999; Marrus et al. 2004; Featherstone et al. 2005; Qin et al. 2005) . Null mutations in the DGluRIIA gene ( *GluRIIA ^AD9^* and *GluRIIA ^SP16^* ) were created through imprecise P-element excisions (Petersen et al. 1997) . Both null alleles generate viable animals which have reduced quantal size as assessed via miniature excitatory postsynaptic potential (mEPSP) amplitudes, but exhibit homeostatic compensation as evidenced by increased quantal content, which works to restore the evoked excitatory postsynaptic potential (EPSP) amplitude (Petersen et al. 1997) . We endeavored to confirm the previously identified bounds of the *GluRIIA ^SP16^* lesion and characterize new *GluRIIA* -specific mutants generated by CRISPR-mediated mutagenesis.

For *GluRIIA ^SP16^* , we undertook a PCR-based approach. We utilized previously identified primers to generate a long (~3 kbp) PCR amplicon spanning the residual P{lacW} element used in the original mutagenesis (Petersen et al. 1997; Brusich 2015) . Sequence analysis of w ^*^ ; *GluRIIA ^SP16^* reconfirmed a lesion spanning from the upstream *Oscillin* gene through exon 9 of *GluRIIA* ( Fig. 1A ). Sequence analysis also confirmed a section of 2,767bp of DNA corresponding to the original *P{lacW}* element used to generate the *GluRIIA ^SP16^* allele was left behind during the imprecise excision (Petersen et al. 1997) . Exact genomic DNA break-points were revised from previous work and span a region of 9,641bp in the reference genome (chr2L: 5,547,701- 5,557,341) (Brusich 2015) .

The knowledge of break-points in *GluRIIA ^SP16^* and of residual P{lacW} material allowed us to revise a multiplex PCR strategy for simple positive identification of heterozygous *GluRIIA ^SP16^* backgrounds (Brusich 2015) . Typically, recombination of *GluRIIA ^SP16^* with another 2 ^nd^ chromosome genetic element has been confirmed via electrophysiological analysis or failure to generate a PCR amplicon in the *GluRIIA* gene region in homozygous animals. However, this process is slow and has risks of false negatives from PCR. We developed two primer pairs for control chromosomes that generate amplicons within the region excised by the *GluRIIA ^SP16^* lesion (501 bp or 712 bp). Either control primer pair can be combined with a primer set which generates an amplicon from genomic DNA across the 5’ break-point to the residual P{lacW} of the *GluRIIA ^SP16^* lesion (243 bp). Thus, via a single PCR reaction and resolution by gel electrophoresis, it is possible to positively identify each of the intact *GluRIIA* gene on a control chromosome and the *GluRIIA ^SP16^* lesion chromosome.

The *GluRIIA* -targeting CRISPR construct ( *sgRNA-GS00444* ) was used to generate mutations in exon 6 of *DGluRIIA* ( Fig. 1A ). Putative mutant fly lines were established and preliminarily screened via electrophysiology of spontaneous miniature neurotransmission. Homozygous *GluRIIA ^SP16^* flies were used as positive controls and exhibited reduced mEPSP amplitudes ( Fig. 1B and 1C) and reduced mEPSP frequencies ( Fig. 1B and 1D) compared to *w ^1118^* controls. Three lines, *GluRIIA ^304^ , GluRIIA ^306^ ,* and *GluRIIA ^704^* , similarly exhibited reduced mEPSP amplitudes and frequencies compared to *w ^1118^* controls (Figs. 1B-D). Each genotype was further subjected to analysis of evoked neurotransmitter release. By our analysis, each of *GluRIIA ^SP16^* , *GluRIIA ^304^ , GluRIIA ^306^ ,* and *GluRIIA ^704^* maintained EPSP amplitudes and had increased quantal content compared to the *w ^1118^* control (Figs. 1B, 1E, and 1F). The NMJ of each putative mutant was dissected and immunostained with anti-GluRIIA antibodies. None of the three lines generated an anti-GluRIIA immunofluorescence signal ( Fig. 1G ), indicating that they are all likely to be *GluRIIA* null alleles.

Each of the three new *GluRIIA* mutants capably produces homozygous, straight-winged adult flies that were used for PCR and sequencing. PCR and sequencing identified mutations in exon 6 of *GluRIIA* targeted by CRISPR-mediated mutagenesis for each of the *GluRIIA ^304^* , *GluRIIA ^306^* , and *GluRIIA ^704^* lines ( Fig. 1H ). Wild-type *GluRIIA* mRNA is predicted to form a 907-amino acid protein (mRNA: NM_078760.3; protein: NP_523484.2). By contrast, each of the CRISPR-generated lines are predicted to have normal mRNA until frame-shifts are introduced at codons that typically encode for Asn272 ( *GluRIIA ^306^* ) or Iso273 ( *GluRIIA ^304^* and *GluRIIA ^704^* ), after which aberrant sequence is predicted until premature stop codons at positions 278 ( *GluRIIA ^304^* ) , 359 ( *GluRIIA ^306^* ), or 284 ( *GluRIIA ^704^* ).

Our results defined the exact bounds of the lesion in the commonly used *GluRIIA ^SP16^* allele ( Fig. 1A ), as well as new lesions in three CRISPR-generated *GluRIIA* mutant lines ( Fig. 1H ). We further revised a multiplex PCR strategy for positive identification of heterozygous *GluRIIA ^SP16^* backgrounds. We found that the new CRISPR-generated *GluRIIA* mutants exhibited reduced mEPSP amplitude and frequency (Figs. 1C and 1D), but normal EPSP amplitudes ( Fig. 1E ) due to robust homeostatic upregulation of quantal content ( Fig. 1F ). Our observation of reduced mEPSP amplitude in the *GluRIIA* mutants was expected and consistent with other *GluRIIA* lesions, expression of dominant negative *GluRIIA* constructs, and RNAi-mediated approaches (Petersen et al. 1997; Diantonio et al. 1999; DiAntonio 2006; Frank 2014; Frank et al. 2020) . It is also consistent with a more recent CRISPR-based effort by a separate group that generated *GluRIIA* indel mutants, including the *GluRIIA ^PV3^* lesion that is predicted to result in a premature stop codon (D277stop) nearly identical to our *GluRIIA ^304^* allele (Goel et al. 2022) .

More interesting is our observation here of homeostatic potentiation that restores the EPSP to that of controls in *GluRIIA ^SP16^* and each of the *GluRIIA ^304^* , *GluRIIA ^306^* , and *GluRIIA ^704^* lines. The *GluRIIA ^SP16^* mutant reliably elicits homeostatic potentiation of quantal content. Often, this compensation maintains the EPSP at control type levels (Petersen et al. 1997; Tsurudome et al. 2010; Gratz et al. 2019; Perry et al. 2022) , but sometimes the elevated quantal content is insufficient to fully maintain the EPSP (Frank et al. 2006; Frank et al. 2009; Müller et al. 2011; Younger et al. 2013; Brusich et al. 2015; Spring et al. 2016; Yeates et al. 2017; James et al. 2019). Subtle differences in recording conditions may contribute to differences in long-term maintenance of homeostatic compensation in *GluRIIA ^SP16^* , as elevation of extracellular calcium is capable of taking the EPSP in a *GluRIIA ^SP16^* background from partially restored to matching that of controls (Frank et al. 2006) . However, genetic background effects may also be at play in these lines which are kept as isolated lab stocks. Differences in activity or expression of a number of presynaptic or postsynaptic factors necessary for long-term homeostatic compensation could be responsible (Frank 2014) . For example, presynaptically, Ca _V_ 2-type calcium channels or IP _3_ receptors which contribute to intracellular calcium, Rab3-GAP and snapin which contribute to vesicle regulation, and K _V_ 4 (shal) ion channel activity are possibilities (Frank et al. 2009; Bergquist et al. 2010; Müller et al. 2011; Dickman et al. 2012). A more intriguing possibility is related to the nature of the *GluRIIA ^SP16^* allele which includes approximately 2,700bp of remnant P{lacW} sequence. In the *GluRIIA ^SP16^* mutant, *GluRIIA* mRNA downstream of the lesion is expressed by an unknown promoter and limited translation occurs as evidenced by ribosome profiling (Chen and Dickman 2017) . Remnant P{lacW} sequence should not be discounted as the possible source of such a promoter (Druker et al. 2004; LaFave and Sekelsky 2011) . Any protein expression of the C-terminal region of GluRIIA has the potential to disrupt full homeostatic scaling as it is capable of sequestering CaMKII and occluding presynaptic homeostatic potentiation (Perry et al. 2022) . Thus, genetic differences which cause changes in transcriptional activity of the residual *GluRIIA* gene or the efficiency of translation may alter the extent to which homeostatic potentiation is actively promoted. We do not know if the new CRISPR-generated mutants have similar, limited expression of the 3’ region of *GluRIIA* and this question may be of interest to future investigations.

## Methods

**Fly Husbandry, Stocks, and CRISPR-mediated mutagenesis**

Control strain *w ^1118^* (Hazelrigg et al. 1984) and w ^*^ ; GluRIIA ^SP16^ (Petersen et al. 1997) stocks are maintained as lab stocks (CAF). Flies were reared at 25°C in temperature- and light-controlled incubators on media containing water, agar, molasses, yellow cornmeal, and yeast throughout CRISPR-mediated mutagenesis crossing steps (Mallik et al. 2017; Brusich et al. 2018) . Flies were housed in similar conditions on water, agar, glucose, yellow cornmeal, yeast, tegosept, and Acid A containing food ahead of DNA isolation steps (Putnam et al. 2019) .

The *GluRIIA ^704^* , *GluRIIA ^304^* ^,^ and *GluRIIA ^306^* alleles were generated by CRISPR-mediated mutagenesis. Flies carrying the *sgRNA-GS00444* element targeting the 5’- CAATCGCACCGACGTAATGTTGG-3’ sequence of Exon 6 of *GluRIIA* (BDSC 68059: y[1] sc[*] v[1] sev[21]; P{y[+t7.7] v[+t1.8]=TKO.GS00444}attP40 ) were crossed to *vas-Cas9* males (BDSC 51324: *w[1118]; PBac{y[+mDint2] GFP[E.3xP3]=vas-Cas9}VK00027* ). Resultant F1 progeny possessing both the *sgRNA* and *vas-Cas9* elements were then crossed to males carrying a 2 ^nd^ chromosome balancer ( *CyO-GFP* ). Last, F2 progeny were selected for putative mutations in *GluRIIA* and against *sgRNA* and *vas-Cas9* chromosomes, and balanced with *CyO-GFP.* Full details and a schematic of the crossing scheme are available upon request.

**Electrophysiology**

*GluRIIA* mutations within putative stocks were initially identified via an electrophysiological approach. All dissections and recordings were performed in modified HL-3 saline (Stewart et al. 1994) containing 70 mM NaCl, 5 mM KCl, 10 mM MgCl2, 10 mM NaHCO3, 115 mM sucrose, 4.2 mM trehalose, 5 mM HEPES, and 0.5 mM CaCl2, pH 7.2. Neuromuscular junction sharp electrode (electrode resistance between 20-30 MΩ) recordings were performed on muscles 6/7 of abdominal segments A2 in wandering third-instar larvae as described (Mallik and Frank 2022) . Recordings were performed on a Leica microscope using a 10x objective and acquired using an Axoclamp 900A amplifier, Digidata 1440A acquisition system, and pClamp 10.7 software (Molecular Devices). Electrophysiological sweeps were digitized at 10 kHz and filtered at 1 kHz. Thirty EPSPs were evoked at each muscle and the average of the 30 sweeps at each muscle were used in analysis and in plotting representative data. Data were analyzed using Clampfit (Molecular Devices) and MiniAnalysis (Synaptosoft) software. Miniature excitatory postsynaptic potentials (mEPSPs) were recorded without any stimulation. A minimum of 4 muscles from across at least 2 animals were used for mEPSP analysis. Stocks with larval NMJs with significant reductions in mEPSP amplitude were identified as putative *GluRIIA* loss-of-function mutant stocks and subjected to further tests.

**Confocal imaging**

Samples were imaged using a Zeiss LSM 710 laser scanning confocal microscope equipped with 63×/1.4 NA oil immersion objective with four laser lines (405, 488, 561, and 637 nm) at room temperature. For fluorescence quantifications, the samples were stained with mouse anti- *GluRIIA* (DSHB: 8B4D2; 1:30), Alexa 488 goat anti-HRP (Jackson ImmunoResearch Laboratories: AB_2338965; 1:400) and goat anti-mouse TRITC (Invitrogen: A16071; 1:200) antibodies in the same tube with similar reagents. The samples were mounted in Vectashield (Vector Laboratories) and imaged in the same session. Z-stacks were obtained using identical settings with z-axis spacing between 0.2-0.5 μm and optimized for detection without signal saturation (Raut et al. 2017) . GluRIIA and HRP-positive boutons were used to analyze the control sample; however, the mutants did not have any signals for GluRIIA. Images were cropped and the brightness and contrast adjustments were applied to signal for GluRIIA in the same way for each image (ImageJ 1.47v).

**DNA Isolation, PCR, Sequencing, and Primers**

Two, homozygous straight-winged male flies of each genotype were used for crude DNA extractions prior to PCR (Engels et al. 1990; Gloor and Engels 1991) . Briefly, the procedure involved homogenization of flies using a micropestle in 50 μL of ‘squishing buffer’ (10 mM Tris-HCl at pH 8.0, 1 mM EDTA, 25 mM NaCl, and 200 μg/mL Roche proteinase K (Fisher Scientific), incubation at 37°C for 30 minutes, heat-inactivation of the proteinase K by holding samples at 95°C for 3 minutes, and removal of the supernatant containing the crude DNA extract.

One μL of crude DNA extract was used for each PCR reaction (25 or 50 μL volume). Primers were used at a final concentration of 0.5 μM, except for multiplex reactions in which primer pairs used to generate shorter amplicons from *GluRIIA ^SP16^* chromosomes were used at 0.25 μM. Phusion High-Fidelity PCR Master Mix with HF Buffer (New England Biolabs) was used for all PCR using the manufacturer’s recommendations except for a long (90 seconds) amplification for reactions across the lesion site in w*; GluRIIA ^SP16^ flies, which generated a ~3 kbp amplicon. The Monarch® PCR & DNA Cleanup Kit (New England Biolabs) was used to purify the PCR product. Single-stranded Sanger sequencing was performed by ACGT Inc. (Wheeling, IL). DNA preparations, PCR, and sequencing were conducted with two separate biological replicates for each genotype.

Primers used for PCR and sequencing of CRISPR-generated mutants and *w ^1118^* generated a 923bp amplicon (5’-CATATGTTCGCGATGTGACG-3’ and 5’-CATCCAAGGAGACGAACTGC-3’). Primers used for PCR and sequencing of *GluRIIA ^SP16^* generated a ~3kbp amplicon from *GluRIIA ^SP16^* (5’-ACACACACGAAAAACACAACTGAAT-3’ and 5’-CTATGAAAACAAAAGCCAAAGTCAT-3’). Multiplex primers for detection of a nonmutated *GluRIIA* gene generated a 501bp amplicon (5’-GACTTTAAGTTGCAGGGCCC-3’ and 5’-CGGTCTTAAAGTGCGGTTCC-3’) or 712bp amplicon (5’-AGTGGATGTAAGTGGAGGCC-3’ and 5’-CATCCCATTGCTTCGTCTCC-3’), and were compatible with primers used for positive detection of the *GluRIIA ^SP16^* allele that generated a 243bp amplicon (5’- AACACAACTGAATGAGCCGC-3’ and 5’- ACAACCTTTCCTCTCAACAAGC-3’)

**Sequence Alignment and Analysis**

Sequence alignment and analysis were performed using SnapGene® (SnapGene®, v6.0.5, Insightful Science). Forward and reverse reads from *w ^1118^* , *GluRIIA ^304^* , *GluRIIA ^306^* , *GluRIIA ^704^* were aligned using the Needleman-Wunsch global alignment method (Needleman and Wunsch 1970) . Poorly aligned sequences at the ends of the reads were trimmed and individual discrepancies were resolved with reference to the chromatogram data. A minimum of 694 bp of perfectly aligned consensus sequence was generated for each genotype. Consensus reads were aligned with genomic DNA from the GluRIIA gene region (BDGP Release 6 + ISO1 MT) using the MUSCLE algorithm (Edgar 2004a; Edgar 2004b). This process was conducted for each of the two biological replicates.

Sequencing forward reads from 2 biological replicates of w ^*^ ; *GluRIIA ^SP16^* were aligned using the Needleman-Wunsch method (Needleman and Wunsch 1970) , and the same was done with reverse reads. Poorly aligned sequences at the ends of the reads were trimmed and individual discrepancies were resolved with reference to the chromatogram data. A minimum of 849 bp of perfectly aligned consensus sequence was generated for each forward and reverse reads. Aligned reads were then aligned with genomic DNA (BDGP Release 6 + ISO1 MT) using the Smith-Waterman local alignment algorithm (Smith and Waterman 1981) . Aligned forward and reverse reads were also aligned to P[lacW] genetic sequence (FlyBase: FBtp0000204) using the Needleman-Wunsch method (Needleman and Wunsch 1970) .
